# Epigenetic Signatures at AQP3 and SOCS3 Engage in Low-Grade Inflammation across Different Tissues

**DOI:** 10.1371/journal.pone.0166015

**Published:** 2016-11-08

**Authors:** Carola Marzi, Lesca M Holdt, Giovanni Fiorito, Pei-Chien Tsai, Anja Kretschmer, Simone Wahl, Simonetta Guarrera, Daniel Teupser, Tim D. Spector, Licia Iacoviello, Carlotta Sacerdote, Konstantin Strauch, Serene Lee, Wolfgang E. Thasler, Annette Peters, Barbara Thorand, Petra Wolf, Holger Prokisch, Rosario Tumino, Christian Gieger, Vittorio Krogh, Salvatore Panico, Jordana T. Bell, Giuseppe Matullo, Melanie Waldenberger, Harald Grallert, Wolfgang Koenig

**Affiliations:** 1 Research Unit of Molecular Epidemiology, Institute of Epidemiology II, Helmholtz Zentrum München, German Research Center for Environmental Health Neuherberg, Germany; 2 German Center for Diabetes Research (DZD e.V.), Neuherberg, Germany; 3 Institute of Laboratory Medicine, Ludwig-Maximilians-University Munich, Munich, Germany; 4 Human Genetics Foundation (HuGeF)–Torino, Turin, Italy; 5 Medical Sciences Department, University of Turin, Turin, Italy; 6 Department of Twin Research and Genetic Epidemiology, King’s College London, London, United Kingdom; 7 Department of Epidemiology and Prevention, IRCCS Istituto Neurologico Mediterraneo Neuromed, Pozzilli (IS), Italy; 8 Unit of Cancer Epidemiology, Citta' della Salute e della Scienza Hospital-University of Turin and Center for Cancer Prevention (CPO), Torino, Italy; 9 Institute of Genetic Epidemiology, Helmholtz Zentrum München, German Research Center for Environmental Health, Neuherberg, Germany; 10 Institute of Medical Informatics, Biometry and Epidemiology, Chair of Genetic Epidemiology, Ludwig-Maximilians-Universität, Munich, Germany; 11 Department of Surgery, Ludwig-Maximilians-Universität München, Munich, Germany; 12 Institute of Epidemiology II, Helmholtz Zentrum München, German Research Center for Environmental Health, Neuherberg, Germany; 13 Institute of Human Genetics, Helmholtz Zentrum München, German Research Center for Environmental Health, Neuherberg, Germany; 14 Institute of Human Genetics, Technical University Munich, München, Germany; 15 Cancer Registry and Histopathology Unit, “Civile–M.P. Arezzo” Hospital, ASP 7, Ragusa, Italy; 16 Epidemiology and Prevention Unit, Fondazione IRCSS Istituto Nazionale Tumori, Milan, Italy; 17 Department of Clinical and Medicine and Surgery, Federico II University, Naples, Italy; 18 Department of Internal Medicine II-Cardiology, University of Ulm Medical Center, Ulm, Germany; 19 Deutsches Herzzentrum München, Technische Universität München, Munich, Germany; 20 DZHK (German Center for Cardiovascular Research), partner site Munich Heart Alliance, Munich, Germany; Centre de Recherche en Cancerologie de Lyon, FRANCE

## Abstract

**Background:**

Elevated levels of C-reactive protein (CRP, determined by a high-sensitivity assay) indicate low-grade inflammation which is implicated in many age-related disorders. Epigenetic studies on CRP might discover molecular mechanisms underlying CRP regulation. We aimed to identify DNA methylation sites related to CRP concentrations in cells and tissues regulating low-grade inflammation.

**Results:**

Genome-wide DNA methylation was measured in peripheral blood in 1,741 participants of the KORA F4 study using Illumina HumanMethylation450 BeadChip arrays. Four CpG sites (located at *BCL3*, *AQP3*, *SOCS3*, and cg19821297 intergenic at chromosome 19p13.2, P ≤ 1.01E-07) were significantly hypomethylated at high CRP concentrations independent of various confounders including age, sex, BMI, smoking, and white blood cell composition. Findings were not sex-specific. CRP-related top genes were enriched in JAK/STAT pathways (Benjamini-Hochberg corrected P < 0.05). Results were followed-up in three studies using DNA from peripheral blood (EPICOR, n = 503) and adipose tissue (TwinsUK, n = 368) measured as described above and from liver tissue (LMU liver cohort, n = 286) measured by MALDI-TOF mass spectrometry using EpiTYPER. CpG sites at the *AQP3* locus (significant p-values in peripheral blood = 1.72E-03 and liver tissue = 1.51E-03) and the *SOCS3* locus (p-values in liver < 2.82E-05) were associated with CRP in the validation panels.

**Conclusions:**

Epigenetic modifications seem to engage in low-grade inflammation, possibly via JAK/STAT mediated pathways. Results suggest a shared relevance across different tissues at the *AQP3* locus and highlight a role of DNA methylation for CRP regulation at the *SOCS3* locus.

## Introduction

Low-grade inflammation is thought to induce, promote or more generally influence human susceptibility to many age-related disorders such as coronary heart disease[1], type 2 diabetes[2], and several malignancies[3]. Modestly elevated concentrations of C-reactive protein (CRP), measured by a high-sensitivity assay, are a sensitive marker of low-grade inflammation. CRP is released into the systemic circulation in response to inflammatory stimuli as the final product of various inflammatory pathways. As an acute-phase reactant it is predominantly synthesized by hepatocytes and regulated via the transcription factors STAT3, C/EBP family members and NF-kappaB by the pro-inflammatory cytokines IL-6 and IL-1ß[4,5]. To a minor degree, extra-hepatic expression has been reported for adipose tissue and blood cells[5]. Systemic levels of CRP are known to be influenced by age, sex, environmental and life style conditions like smoking exposure and BMI as well as genetic determinants with substantial heritability estimates[6]. Recent research focusing on common sequence variants has only partially explained the molecular basis of systematically circulating CRP[7].

Epigenetic modifications such as changes in DNA methylation seem to have important regulatory functions in cellular processes[8], including inflammatory responses of the human body[9]. Previous studies suggested that non-genetic determinants of CRP like age[10–12], sex[13,14], diet[15] and exposure to cigarette smoking[16] as well as genetic factors[17,18] are associated with epigenetic modification. In addition, evidence indicated that epigenetic mechanisms are implicated in the development of several malignancies[19] as well as atherosclerosis[20] which are both characterized by aberrant inflammatory processes[21,22]. Finally, epigenetic modifications were observed in several inflammatory disease states[23–27]. Therefore, epigenetic modifications integrating both environmental as well as genetic factors might relevantly engage in low-grade inflammation as reflected by elevated levels of circulating CRP.

Epigenome-wide association studies (EWAS) on DNA methylation hold the potential to identify epigenetic modifications of CRP regulation across the genome. This could provide important clues to immune response pathways involved in the regulation of low-grade inflammation and might also be of relevance for related clinical entities. Therefore, we conducted an EWAS on CRP concentrations in a large population-based study using DNA methylation data in peripheral blood. To validate and to assess tissue specificity and relevance of the discovery findings, results were followed up in three independent studies using DNA methylation data derived from peripheral blood, adipose and liver tissue. In addition, gene expression panels for validated genes were generated, transcript levels of these genes were quantified in human liver samples, and enrichment analyses were conducted to ascertain functional properties of identified loci.

## Results

### Discovery of DNA methylation sites related to CRP in peripheral blood

**Fig 1:**
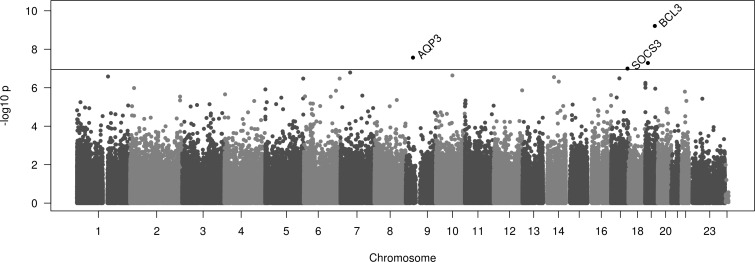
Manhattan Plot of the results of the genome-wide DNA methylation analysis on CRP conducted in the KORA F4 discovery sample. The Manhattan plot displays all analyzed CpG sites with their calculated p-values. Threshold of epigenome-wide significance: P = 1.13E-07.

We observed epigenome-wide significant associations with systemic CRP concentrations for 4 CpG sites located intronic at *B-cell lymphoma 3 (BCL3)*, and *aquaporin 3 (AQP3)*, in exon 2 at *Suppressor of cytokine signaling (SOCS3)*, and intergenic (12kb upstream of jun B proto-oncogene, *JUNB*) at chromosome 19p13.2 in the comprehensive model (p-values range 6.14E-10–1.01E-07, Table 1, Fig 1). There was an inverse association between DNA methylation and levels of CRP for all significant CpG sites with effects ranging from -0.023 to -0.031.

**Table 1:** Significant associations between CRP and DNA methylation sites in the discovery and validation panels.

Locus			KORA F4			EPICOR			TwinsUK			LMU liver cohort		
Gene	chr	CpG	ß coef	se	p	ß coef	se	p	ß coef	se	p	ß coef	se	p
*BCL3*	19	cg26470501	-0.03	0.005	**6.14E-10**	-0.02	0.013	1.21E-01	-0.02	0.01	3.12E-01	-0.01	0.01	3.24E-01
*AQP3*	9	cg02716826	-0.03	0.005	**2.72E-08**	-0.04	0.011	**1.72E-03**	-0.02	0.01	2.44E-01	0.04	0.01	**1.51E-03**
NA*	19	cg19821297	-0.02	0.004	**5.19E-08**	-0.02	0.01	6.56E-02	-0.001	0.01	9.01E-01	-0.02	0.01	8.62E-03
*SOCS3*	17	cg18181703	-0.02	0.004	**1.01E-07**	-0.01	0.011	1.89E-01	-0.01	0.01	4.27E-01	-0.01	0.01	1.12E-01
*SOCS3*	17	CpG_2.3	NA	NA	NA	NA	NA	NA	NA	NA	NA	-0.04	0.01	**4.36E-07**
*SOCS3*	17	CpG_8.9	NA	NA	NA	NA	NA	NA	NA	NA	NA	-0.03	0.01	**2.82E-05**

Significant associations between ln-transformed systemic CRP levels and beta values of DNA methylation sites were assessed using multivariate linear mixed effects models in KORA F4 (n = 1741), EPICOR (n = 503), and TwinsUK (n = 286) and multivariate linear models in the LMU liver cohort (n = 286) adjusting for various confounding variables and correcting for multiple testing according to Bonferroni. Significant p-values (1.13E-07, 1.25E-02, and 1.92E-03 in KORA F4, EPICOR and TwinsUK and the LMU liver cohort, respectively) are marked in bold font. chr: chromosome; Gene: UCSC reference gene according to USCS Genome Browser; ß coef: β coefficient; se: standard error

*no gene annotation for this CpG site according to the UCSC Genome Browser.

No statistically significant interaction term between methylation beta values of the 4 significant CpG sites and sex was observed (data not shown).

We identified correlations ≥ 0.8 with SNPs (listed in Table A in S1 File) for two CpG sites (cg26470501 and cg19821297). Associations remained similar when we repeated the analyses for these two loci with additional adjustment for correlated SNPs in participants of the KORA F4 study where genomic and DNA methylation data were available (Table A in S1 File). Likewise, associations remained within similar ranges when we repeated the analyses with additional adjustments for lipids, uric acid, leptin, fasting glucose, alcohol consumption, systolic blood pressure, or medication (Table A in S1 File).

### Enrichment analysis

All unique genes identified in the EWAS at a 5% false discovery rate significance level (23 genes, File A and Table B in S1 File) were annotated in the Ingenuity Pathway Analysis (IPA) database. The enrichment analysis yielded 6 statistically significant canonical pathways with Benjamini-Hochberg corrected p-values < 0.05 (Table 2).

**Table 2:** Significant canonical pathways in the KOFA F4 discovery panel.

Canonical pathways	Benjamini-Hochberg p-value	Genes
Role of JAK2 in Hormone-like Cytokine Signaling	0.02	*SOCS3*, *SH2B2*
IL-9 Signaling	0.02	*SOCS3*, *BCL3*
Role of JAK1 and JAK3 in Cytokine Signaling	0.04	*SOCS3*, *SH2B2*
Growth Hormone Signaling	0.04	*SOCS3*, *RPS6KA2*
Acute Myeloid Leukemia Signaling	0.04	*RARA*, *PIM2*
Melanocyte Development and Pigmentation Signaling	0.04	*RPS6KA2*, *SH2B2*

### Replication of significant loci in peripheral blood

One of the four significant CpG sites (cg02716826 at *AQP3*) was confirmed in the EPICOR peripheral blood cohort with the effect estimate being consistent with the one of the discovery analysis in direction and magnitude of effect (Table 1). The CpG site cg02716826 remained statistically significant after further adjustment for myocardial infarction case-control status (ß coefficient = -0.035, se = 0.011, p = 2.13E-3).

### Validation of significant loci in adipose tissue and in liver samples

In the TwinsUK study, associations were consistent with those of the discovery analysis in direction and magnitude of effects but p-values were not statistically significant (Table 1).

In the LMU liver cohort, CpG sites representing 2 of the discovery loci (*AQP3* and *SOCS3*) yielded statistically significant results in the validation analyses (Table 1). At the *AQP3* locus the discovery CpG site cg02716826 was significantly associated with CRP. At the *SOCS3* locus the discovery CpG site cg18181703 was not associated with CRP but adjacent CpG sites which were significantly correlated with the discovery CpG site (Spearman’s rank correlation coefficients = 0.48 and 0.52 for CpG_2.3 and CpG_8.9, respectively, each with P = 2.2E-16) yielded statistically significant associations. A full table of results of the LMU liver cohort is presented in Table C in S1 File.

### Results of gene expression analyses

In the gene expression panels, both genes, *AQP3* and *SOCS3*, were expressed not only in blood cells but also to different degrees in human tissues (Fig A in S1 File). While *AQP3* was mainly expressed in kidney, lung, heart, and liver tissue, *SOCS3* displayed highest gene activity in heart and adipose tissue.

In the LMU liver cohort, gene expression levels of *SOCS3* were significantly correlated with CRP concentrations (Spearman’s rank correlation coefficient = 0.15, P = 0.01) but not with cg18181703 or any other CpG site covered by the corresponding amplicon (data not shown). Gene expression levels of *AQP3* were not correlated with CRP concentrations but displayed a significant correlation with CpG_5 of the corresponding amplicon (Spearman’s rank correlation coefficient = -0.14, P = 0.01).

## Discussion

In this study we report key findings from an epigenetic study on low-grade inflammation as reflected by concentrations of CRP in cells and tissues regulating low-grade inflammation.

### DNA methylation sites were associated with CRP in peripheral blood independent of other risk factors

In a first step, we conducted an EWAS in a large population-based study and identified four loci at chromosomes 9, 17 and 19 inversely associated with concentrations of CRP. A lower degree of DNA methylation at some CpG sites was also observed in conditions related to elevated levels of CRP such as older age[12], adiposity[28], and smoking[16,29,30]. However, in the present study the association was independent of various CRP determinants including age, sex, BMI, and cigarette smoking. A general inverse association is consistent with findings of a previous EWAS conducted in hypertensive African Americans using DNA methylation data derived from leucocytes[31].

### Functional properties of inflammation-related epigenetic signatures in peripheral blood

Six statistically significant pathways were identified in enrichment analyses including top CRP related genes identified in the EWAS. Janus kinase (JAK)/ Signal Transducer and Activator of Transcription (STAT) signaling plays a major role in almost all of these pathways. The highly conserved JAK/STAT signaling pathway is part of the orchestrated cascade during the acute-phase response transmitting extra-cellular signals through the cell membrane into gene promoters[32]. Thereby, it modulates transcription of genes like the acute-phase proteins fibrinogen, serum amyloid A, and CRP which are all responsive to STAT3. The importance of JAK/STAT signaling in the regulation of the immune system was further highlighted by studies reporting immune deficiency syndromes following disruption or dysregulation of JAK/STAT functionality[33]. Thus, from a biological perspective a conceivable mechanism via JAK/STAT signaling seems to be plausible.

### DNA methylation signatures at AQP3 and SOCS3 were associated with CRP across different tissues

In order to validate the discovery findings as well as to assess whether the identified epigenetic effects are specific or shared across different tissues relevant for CRP expression results were followed up in three independent studies using DNA methylation data derived from peripheral blood, liver and adipose tissues. In these tissue-specific analyses we found significant associations at two loci, *AQP3* and *SOCS3*. Both genes seem to be active in different human tissues according to our gene expression panels.

At the *AQP3* locus, associations between DNA methylation and CRP were statistically significant in peripheral blood and liver tissue. In addition, we observed a significant correlation between DNA methylation at *AQP3* and *AQP3* transcript levels in human liver samples. Main functions of the aquaporin 3 protein are the transport of water, glycerol and small solutes such as urea and glycerol across the plasma membrane but it also seems to be involved in functions related to the immune system like wound healing[34] and the activation of the skin immune system at birth[35]. Furthermore, an up-regulation of *AQP3* was observed under chronic inflammatory conditions such as present in periodontitis[36] and gastritis[37]. Interestingly, at cg02716826 which was measured in all four studies CRP effects were negative in the peripheral blood samples as well as in adipose tissue while they were positive in liver tissue. A complex and tissue specific relationship was also observed in different cell line studies investigating *AQP3* expression in response to cytokine signaling. In one of these studies, using gingival epithelial cells gene expression was increased in response to TNF-alpha[36] while other studies report decreased expression levels in response to TNF-alpha in keratinocytes[38,39]. Our study seems to confirm tissue specific effects at this locus. However, as BMI or other measures of adiposity were not available in the liver study, we cannot exclude that effect differences might be caused by confounding. Hence, epigenetic mechanisms at the *AQP3* locus seem to have a shared relevance in low-grade inflammation across different tissues, but further studies are warranted to evaluate tissue specificity of effects and the influence of adiposity in liver tissue.

The second region at *SOCS3* was significantly associated with systemic levels of CRP in the peripheral blood discovery analysis as well as in the liver samples. Furthermore, *SOCS3* transcript levels were significantly correlated with CRP in human liver tissue. SOCS3 is a negative feedback regulator of cytokine signaling along the JAK/STAT pathway. Growing evidence supports a role of epigenetic mechanisms at *SOCS3* in several cancers including liver, lung, pancreatic and prostate cancer, as well as other malignancies[40–45]. Given well confirmed evidence of the presence of inflammatory cells in tumor microenvironment[3,21] our finding supports a role of epigenetic mechanisms at *SOCS3* in conditions related to inflammation and suggests a novel link between DNA methylation at *SOCS3* and systemic CRP in tissues in which immune mediators are expressed.

As the approach taken in this study is observational in nature it is not possible to draw causal inferences. Therefore, it might be possible that not the genes themselves, but small regulatory elements might engage in the identified associations between epigenetic modifications and CRP regulation. This is most likely the case as there has been evidence of regulatory elements like relevant transcription factor binding sites such as C/EBP and NF-kappaB as well as STAT3 in the *AQP3* and the *SOCS3* region, respectively (http://genome.ucsc.edu). The identification of causal inferences and molecular pathways underlying the relation between CpG sites and CRP levels represents promising targets for future functional studies.

### Limitations and strengths

Two limitations of our study have to be mentioned. Firstly, epigenetic modifications are cell-type specific and peripheral blood constitutes a heterogeneous admixture of different cell populations. Observed changes in DNA methylation profiles might therefore reflect a differential representation of the cell types in the sample leading to false positive results[46]. However, for low-grade inflammation peripheral blood is a tissue of interest and a valuable source of information. Therefore, we used peripheral blood derived DNA methylation data in the discovery sample and in one of the validation samples. In addition, to diminish the risk of cell type confounding we adjusting the peripheral blood analyses for white blood cell composition estimated using algorithms developed on the basis of cell-type specific DNA methylation markers identified from cell-sorted reference profiles of specific cell populations[47]. Secondly, validation studies exhibited moderate sample sizes as well as differences in study design or availability of adjustment variables. In particular, further studies are warranted to investigate the effect of BMI on DNA methylation in liver tissue and epigenetic modifications in male adipose tissue. However, the present study had enough power to identify novel epigenetic patterns related to low-grade inflammation with a shared relevance across different tissue. Findings were plausible from a biologic perspective, and may promote future research on the regulation of low-grade inflammation and mechanisms contributing to related clinical disorders. The identified epigenetic patterns may be used not only in functional studies to provide further insights into molecular mechanisms of inflammatory processes but also in biomarker studies using whole blood to improve the prediction of inflammation related clinical disorders or events. In addition, gene expression panels suggest further tissues which seem to be relevant for the identified genes and promising for in-depth investigations with respect to clinical disorders affecting these tissues. Thereby, the identified epigenetic loci might present useful targets for the prevention and / or treatment of these diseases.

### Conclusions

Using an epigenetic approach with DNA derived from different trait targeted tissues the present study identified epigenetic loci which seem to engage in low-grade inflammation independent of various confounders and conceivably via JAK/STAT signaling. In addition, validation results suggest novel evidence for an epigenetic mode at *AQP3* with possible tissue specific effects but a shared relevance for CRP regulation and extend previous evidence of the importance of epigenetic modifications at *SOCS3* with respect to inflammatory processes.

## Materials and Methods

### Study design

**Fig 2:**
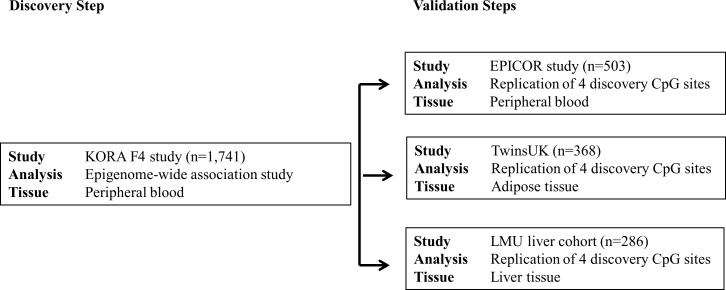
Outline of the present study.

The present study incorporated data from four different studies and followed a two-stage design (Fig 2). In the discovery step, we assessed the association between CRP and the degree of DNA methylation using peripheral blood from 1,741 participants of the population-based Cooperative Health Research in the Region of Augsburg (KORA) F4 study. Subsequent follow-up of results was performed in three independent validation panels: the cardiovascular section of the Italian European Prospective Investigation into Cancer and Nutrition cohort (EPICOR, n = 503), the TwinsUK cohort (TwinsUK, n = 368 female participants), and a cohort from the Ludwig-Maximilians-University Munich (LMU liver cohort, n = 286) using DNA methylation data derived from peripheral blood, adipose, and liver tissue, respectively. For the present study, subjects with elevated levels of CRP indicating acute infection (CRP > 10 mg/L) and/ or missing data on CRP were excluded. All studies were approved by the local ethics committees. In detail, the KORA study and the LMU study were approved by the Ethics Committee of the Bavarian Medical Association (Bayerische Landesärztekammer); the EPICOR study was approved by the Ethical Committee of the Human Genetics Foundation (Turin, Italy); and the TwinsUK study has ethical approval from the Guy’s and St Thomas’ (GSTT) Ethics Committee. Written informed consent was obtained from all participants. Study populations are described in File B in S1 File. Baseline characteristics of participants of the four studies are provided in Table 3.

**Table 3:** Baseline characteristics of study participants.

	KORA F4	EPICOR	TwinsUK	LMU cohort
N (%)	1,741	503	368	286
Age (years) /	60.9 (8.89)	52.5 (7.33)	60.6 (8.04)	
Age (10 yrs)				5.7 (1.36)
Male (%)	48.8	62	0	52.4
BMI (kg/m^2^)	28.1 (4.68)	26.6 (3.89)	26.6 (4.67)	NA
current/former/never smoker (%)	15 / 41 / 44	32 / 31 / 37	10 / 38 / 52	22 / 0 / 61
fasting (%)	99.5	29	NA	100
CRP mg/L*	1.25 (0.62–2.47)	1.07 (0.49–2.19)	1.57 (0.72–3.37)	0.4 (0.2–0.98)

Data with normal and skewed distribution (indicated by *) are given as mean (SD) and median (interquartile range) of the variables.

### Measurement of CRP

CRP was measured in all four studies using high-sensitivity tests (File C in S1 File). The intra- and inter-assay coefficients of variation were below 6% in all four studies.

### Assessment of DNA methylation data

Genome-wide DNA methylation in KORA F4, EPICOR, and TwinsUK was assessed using the Illumina HumanMethylation450 BeadChip. In brief, genomic data was bisulfite converted. Subsequently, the bisulfite converted samples were amplified and after enzymatic fragmentation and application of the samples the arrays were fluorescently stained and scanned. Beta values representing the percentage of DNA methylation of a cytosine were calculated as the ratio of the methylated signal over the sum of the methylated and unmethylated signals. Further details on DNA methylation measurement, data preprocessing, and quality assessments of the four studies are presented in File D in S1 File. Annotations are based on UCSC Genome Browser on Human Feb. 2009 (GRCh37/hg19) Assembly (https://genome.ucsc.edu/).

Replication in the LMU liver cohort samples was carried out by MALDI-TOF mass spectrometry using EpiTYPER by MassARRAY (Sequenom, San Diego, CA)[48]. Four amplicons covering 26 CpG sites were selected. Target regions were amplified at 58°C using the prime pairs described in Table D in S1 File. The chip was read by Sequenom MALDI-TOF MS Compact Unit and visualized using MassARRAY EpiTyper v1.2 software (Sequenom). Beta values were determined by comparing the signal intensities between the mass signals of methylated and non-methylated templates.

### Assessment of gene expression data

In order to assess cell type and tissue specificity of the validated results we generated gene expression panels in which we quantified the expression of *AQP3* and *SOCS3* in different human tissues and blood cell types. Primers and probes for quantitative PCRs (qPCRs) of *AQP3* and *SOCS3* are given in Table E in S1 File. Total RNA (1 μg) from human liver, brain, heart, lung, kidney, small intestine, adipose tissue, skeletal muscle, peripheral blood mononuclear cells (PBMC), CD14-, CD19-, CD3-, CD4-, CD8-positive cells, and regulatory T-cells (pool of 2–3 donors; Clontech) was reverse transcribed and qPCRs were performed in quadruplicate as described[49]. Absolute copies were determined using plasmid standard curves and normalized to μg input RNA. RNA from human livers (n = 304; HTCR Stiftung[50]) was isolated with TRIzol according to the manufacturer’s instructions and 2 μg RNA was reverse transcribed according to published protocols[49]. Normalization was performed using house-keeping gene expression of *ACTB* and *GAPDH[49].* Subjects with DNA methylation data and data on CRP concentrations ≤ 10 mg/L were included (n = 286).

### Statistical analyses

#### Discovery analysis

In KORA F4 analysis natural log-transformed concentrations of CRP were modeled using linear mixed effects models with DNA methylation beta values, age, sex, BMI, fasting status (two categories: fasting for > 8 hours /non-fasting), and cigarette smoking (ever/former/never smoker) as fixed effects and technical variables (plate and position on plate) as random effects. In addition, because peripheral blood constitutes a heterogeneous admixture of different cell types which may be methylated in a cell-type specific way principal components of white blood cell components estimates[47] were added as fixed effects to adjust for cell type confounding.

#### Sensitivity analyses

Sensitivity analyses with various degrees of adjustment were performed and the following covariates were added to the statistical model: lipids (lipid ratio defined as total cholesterol levels divided by high-density lipoprotein cholesterol, triglycerides, and low-density lipoprotein cholesterol), uric acid, leptin, fasting glucose, alcohol consumption [g/day], systolic blood pressure, or systemic hormone therapy (yes/no/male) and other medication including, regular intake of corticoids or non-steroidal anti-inflammatory drugs, antidiabetic medication, intake of antihypertensive and lipid lowering drugs (yes/no).

In addition, effect modifications by sex were assessed by adding an interaction term in the multivariate models.

To control for genomic confounding, correlations between CRP related CpG sites identified in the KORA F4 discovery sample and SNPs with minor allele frequency > 0.05 were assessed using genomic data previously acquired in the KORA F4 study (File B in S1 File). Subsequently, for CpG sites which were correlated with one or more common sequence variants (correlation coefficient ≥ 0.8) the analysis was repeated, this time including principal components derived from the correlated SNPs in the multivariate linear mixed effects models.

#### Enrichment analyses

All CpG sites which were significant at a false discovery rate level in the KORA F4 study were included in pathway analyses. Pathway analyses were performed using the Ingenuity Pathway Analyses (IPA) software tool (IPA build version 338830M, content version: 23814503, release date 2015-03-23, analysis date 2015-04-20; http://www.ingenuity.com/). Gene enrichment in canonical pathways was assessed in the core analysis module using Fisher’s exact test right tailed with Benjamini-Hochberg corrected level of significance.

#### Validation analyses

To replicate the findings in another study using DNA methylation data from peripheral blood, we assessed the association between CRP concentrations and methylation beta values of significant CpG sites in the EPICOR study using the statistical model of the discovery analysis. Study center reflecting the fasting state of participants was used as proxy for fasting status.

To assess tissue specificity of the discovery findings results were also assessed in the TwinsUK study and the LMU liver cohort. In TwinsUK, all subjects were female and some subjects were twin pairs. Therefore, sex was not a covariate but family and zygosity were included as random effects in the model. Furthermore, CRP concentrations were not measured at the time of DNA extraction and the difference in years between measurements was used as fixed effect covariate. TwinsUK data analysis also considered other covariates such as age, BMI, and smoking status (ever/former/never smoker) as fixed effects and technical covariates (plate, bisulfite conversion levels and bisulfite conversion efficiency) as random effects. For the analysis in the LMU liver cohort, natural log-transformed levels of CRP were modeled in a linear model using DNA methylation beta values, 10 years age groups, sex, smoking (yes/no), experimental plate, information on chemotherapy (yes/no), and indication of surgery (metastasis of hepatocellular, cholangiocellular or colorectal carcinomas; other metastasis; benign liver tumor; other) as covariates in the model.

#### Gene expression analyses

Correlations between normalized transcript levels of *AQP3* and *SOCS3* and CRP as well as between normalized transcript levels of *AQP3* and *SOCS3* and CpG sites of corresponding amplicons were assessed using Spearman’s rank correlation coefficient.

#### Multiple testing

Results were corrected for multiple testing and thresholds of significance were adapted according to Bonferroni in all four studies. P-values below 1.13E-07 (KORA F4), 1.25E-02 (EPICOR and TwinsUK), and 1.92E-03 (LMU liver cohort) were considered significant.

All statistical analyses were carried out using the software R version 3.0.2 (http://www.r-project.org/).

## Supporting Information

S1 File Supplementary Material. File A in S1 File. Significant associations (Benjamini Hochberg corrected) between CRP and DNA methylation sites in the KORA F4 discovery study. File B in S1 File. Study Populations. File C in S1 File. Measurement of CRP. File D in S1 File. Assessment of DNA methylation data in KORA F4, EPICOR, and TwinsUK using the Illumina HumanMethylation450 BeadChip. Table A in S1 File. Associations between CRP and DNA methylation sites in the KORA F4 discovery study (n = 1741) after additional adjustments. Table B in S1 File. Significant associations (Benjamini Hochberg corrected) between CRP and DNA methylation sites in the KORA F4 discovery study. Table C in S1 File. Associations between CRP and DNA methylation sites in the LMU liver cohort. Table D in S1 File. Sequences of PCR tagged primers used for EpiTYPER methylation analysis, product size of each amplicon, and informative CpG sites per amplicon. Table E in S1 File. Primers and probes for quantitative PCRs. Figure A in S1 File. Expression of *AQP3 and SOCS3* (normalized to μg input RNA) in different human tissues (human brain, heart, lung, kidney, small intestine, adipose tissue, skeletal muscle) and blood cell types (peripheral blood mononuclear cells (PBMC), CD14-, CD19-, CD3-, CD4-, CD8-positive cells, and regulatory T-cells).
(DOCX)

## Abbreviations

AQP3: Aaquaporin 3
BCL3: B-cell lympohoma 3
CRP: C-reactive protein
EPICOR: the cardiovascular section of the Italian European Prospective Investigation into Cancer and Nutrition cohort
EWAS: Epigenome-wide association studies
hs: high-sensitivity
IPA: Ingenuity Pathway Analyses
JAK: Janus kinase
JUNB: jun B proto-oncogene
KORA: Cooperative Health Research in the Region of Augsburg
LMU: Ludwig-Maximilians-University Munich
SOCS3: Suppressor of cytokine signaling (SOCS3)
STAT: Signal Transducer and Activator of Transcription
