# Supplementary material for: Epigenetic Signatures at AQP3 and SOCS3 Engage in Low-Grade Inflammation across Different Tissues
Source: PLoS One. 2016 Nov 8;11(11):e0166015. doi: 10.1371/journal.pone.0166015 (PMC5100881; doi:10.1371/journal.pone.0166015)
Supplement: S1 File — File A in S1 File. Significant associations (Benjamini Hochberg corrected) between CRP and DNA methylation sites in the KORA F4 discovery study. File B in S1 File. Study Populations. File C in S1 File. Measurement of CRP. File D in S1 File. Assessment of DNA methylation data in KORA F4, EPICOR, and TwinsUK using the Illumina HumanMethylation450 BeadChip. Table A in S1 File. Associations between CRP and DNA methylation sites in the KORA F4 discovery study (n = 1741) after additional adjustments. Table B in S1 File. Significant associations (Benjamini Hochberg corrected) between CRP and DNA methylation sites in the KORA F4 discovery study. Table C in S1 File. Associations between CRP and DNA methylation sites in the LMU liver cohort. Table D in S1 File. Sequences of PCR tagged primers used for EpiTYPER methylation analysis, product size of each amplicon, and informative CpG sites per amplicon. Table E in S1 File. Primers and probes for quantitative PCRs. Figure A in S1 File. Expression of AQP3 and SOCS3 (normalized to μg input RNA) in different human tissues (human brain, heart, lung, kidney, small intestine, adipose tissue, skeletal muscle) and blood cell types (peripheral blood mononuclear cells (PBMC), CD14-, CD19-, CD3-, CD4-, CD8-positive cells, and regulatory T-cells). (DOCX) [file pone.0166015.s001.docx]

# Supplemental Material

## File A. Significant associations (Benjamini Hochberg corrected) between CRP and DNA methylation sites in the KORA F4 discovery study

To assess functional properties of inflammation-related epigenetic signatures in peripheral blood we had a closer look at top related loci identified in the discovery study. In fact, several of these loci were related to genes with known direct or indirect functions in the immune system. For example, *strawberry notch homolog 2* (*SBNO2*) is a component of the IL10 pathway that represses inflammatory gene expression, and *phosphoinositide-3-kinase interacting protein 1* (*PIK3IP1*) is associated with T-cell activation. Other genes are implicated in (de-)activation of CRP related transcription factors such as NF-kappaB (*B-cell lymphoma 3* (*BCL3*), *retinoic acid receptor alpha* (*RARA*), *aryl-hydrocarbon receptor repressor* (AHRR), *Pim-2 proto-oncogene, serine/threonine kinase* (*PIM2*), and *TNFAIP3 interacting protein 2* (*TNIP2*)). Finally, the *suppressor of cytokine signaling 3* (*SOCS3*) is known to be interfering with Janus kinase (JAK)/Signal Transducer and Activator of Transcription (STAT) signaling.

## File B. Study Populations

**KORA F4**

The Cooperative Health Research in the Region of Augsburg (KORA) F4 study (2006-2008) is part of a series of independent population-based epidemiological surveys and follow-up studies conducted in residents of German nationality living in the region of Augsburg[[1](#_ENREF_1)]. For the present EWAS, subjects with elevated levels of CRP indicating acute infection (CRP > 10 mg/L, n = 56) and/ or missing data in CRP (n = 2) were excluded. A total of 1,741 subjects were included in the analysis.

In addition, dependency of the results on correlated common sequence variants was assessed in a subpopulation of 1,674 subjects for which both genome and DNA methylation data were available. Genotypes in these samples were determined using Affymetrix Human SNP Array 6.0 for genotyping, Birdseed2 clustering algorithm for calling, and IMPUTE v.0.4.2 software based on HapMap II for imputation. For quality control purposes, a positive control and a negative control DNA was applied every 96 samples. In addition, we excluded subjects with overall genotyping efficiencies below 93% and discordance between called sex and sex in the KORA study database.

**EPICOR**

The peripheral blood replication study EPICOR is an independent case-control study nested within the European Prospective Investigation into Cancer and Nutrition (EPIC)-Italy cohort[[2](#_ENREF_2)]. Aim of EPICOR is to investigate dietary- and lifestyle-related cardiovascular risk factors in more than 1,500 subjects from 4 Italian recruitment centers (Turin, Varese, Naples, Ragusa). The present study was conducted in 503 participants of a case-control subproject on myocardial infarction as described previously[[3](#_ENREF_3)] after additional exclusion of 67 subjects with missing data on CRP and 14 subjects with CRP > 10 mg/L.

**TwinsUK**

Validation in adipose tissue DNA methylation was pursued in 368 female twins from the TwinsUK cohort[[4](#_ENREF_4)]. Adipose tissue biopsies in these individuals were obtained as part of the Multiple Tissue Human Expression Resource (MuTHER) study[[5](#_ENREF_5)].

**LMU liver cohort**

In the LMU liver cohort human liver and serum samples and annotated data were obtained and experimental procedures were performed within the framework of the nonprofit foundation HTCR[[6](#_ENREF_6)] including informed patient's consent. Analyses were performed in 286 subjects with DNA methylation data and concentrations of CRP ≤ 10mg/L.

## File C. Measurement of CRP

In KORA F4, blood was collected in fasting subjects without stasis and kept at 4°C until centrifugation. Plasma concentrations of CRP were assessed by immunonepheleometry on a BN II analyzer (Siemens, Eschborn, Germany). In EPICOR, CRP was determined in plasma by a latex particle-enhanced immunoturbidimetric assay (IL Coagulation Systems on ACL9000). In TwinsUK, CRP levels were measured in serum using a latex enhanced assay (Boeringher nephlometer) and a highly sensitive automated microparticle capture enzyme immunoassay (Abbott Laboratories). In the LMU liver cohort, CRP was assessed by a turbidimetric assay on an AU 5800 analyzer (Beckman Coulter, Krefeld, Germany).

## File D. Assessment of DNA methylation data in KORA F4, EPICOR, and TwinsUK using the Illumina Human Methylation 45 Bead Chip

For KORA F4, DNA methylation measurement has been described in detail before[[7](#_ENREF_7)]. Briefly, genomic DNA from 1,814 samples was bisulfite converted. After amplification, enzymatic fragmentation, and application of the samples the arrays were fluorescently stained and scanned using Illumina HiScan SQ scanner. GenomeStudio (version 2010.3) with methylation module version 1.8.5 was used to process raw image data and assess data quality. Raw methylation values were pre-processed using R, version 3.0.1. Some preprocessing steps were adopted from the pipeline by Touleimat and Tost[[8](#_ENREF_8)]. Briefly, probes with signals summarized from less than three functional beads, and probes with a detection p-values larger than 0.01 were defined as low-confidence probes. Samples with more than 20% low-confidence probes were removed from the data set. Sites representing or being located in 50 bp proximity to single nucleotide polymorphisms (SNPs) with a minor allele frequency of at least 5% were excluded from the data set. Color bias adjustment using smooth quantile normalization, and background correction based on the negative control probes present on the BeadChip, separately for the two color channels, were conducted using the R package *lumi*, version 2.12.0[[9](#_ENREF_9)]. In addition, beta-values from low-confidence probes were set to missing, and CpG sites with more than 5% low-confidence probes removed from the data[[8](#_ENREF_8)]. Finally, beta-mixture quantile normalization (BMIQ)[[10](#_ENREF_10)] was applied to the DNA methylation data using the R package ‘wateRmelon’, version 1.0.3[[11](#_ENREF_11)].

For EPICOR, after checking the quality and integrity of the DNA, DNA was bisulfite converted (EZ-96 DNA Methylation-Gold Kit, Zymo Research Corporation) and methylation levels assessed according to manufacturer’s instructions. Raw data were quality controlled as follows: single Beta- values with detection p-value ≥ 0.01 were excluded from the analysis, as well as (1) CpG loci with detection p-value ≥ 0.01 in more than 20% of the assayed samples, (2) probes containing SNPs with MAF ≥ 0.05 in the CEPH (Utah residents with ancestry from northern and western Europe, CEU) population, and (3) samples with a global call rate ≤ 95%. Background normalization and correction for batch effects were performed on raw methylation[[12](#_ENREF_12)]. Briefly, signal intensities and detection P- values were generated using GenomeStudio software, without background subtraction and control probe normalization. Color-bias adjustment and quantile normalization were performed on signal intensities as implemented in ‘lumi’ R package[[9](#_ENREF_9)]. Finally, BMIQ on beta-values was performed as implemented in the R package ‘wateRmelon’[[11](#_ENREF_11)].

In the TwinsUK sample, DNA methylation levels were profiled on the Illumina 450k DNA methylation array[[13](#_ENREF_13)]. BMIQ[[10](#_ENREF_10)] normalization was applied to the DNA methylation data in 368 subjects. DNA methylation probes that mapped incorrectly or to multiple locations in the reference sequence were removed. Probes with detection P-value > 0.05 in more than 1% of subjects were also removed. All the probes considered in downstream analyses had no missing values.

## Table A. Associations between CRP and DNA methylation sites in the KORA F4 discovery study (n=1741) after additional adjustments

Additional adjustment for PCs of correlated

SNPs†

lipids‡ uric acid levels leptin levels fasting glucose levels

alcohol consumption [g/day]

systolic blood pressure

medication§

| Gene | chr | CpG | ß | p | ß | p | ß | p | ß | p | ß | p | ß | p | ß | p | ß | p |
| --- | --- | --- | --- | --- | --- | --- | --- | --- | --- | --- | --- | --- | --- | --- | --- | --- | --- | --- |
| *BCL3* | 19 | cg26470501 | -0.03 | 1.36E-10 | -0.03 | 1.08E-10 | -0.03 | 1.20E-08 | -0.03 | 7.40E-10 | -0.03 | 9.33E-10 | -0.03 | 7.84E-10 | -0.03 | 3.88E-10 | -0.03 | 2.19E-09 |
| *AQP3* | 9 | cg02716826 | -0.03 | 2.72E-08 | -0.02 | 1.59E-08 | -0.02 | 1.81E-07 | -0.03 | 4.68E-08 | -0.03 | 3.90E-08 | -0.03 | 3.56E-08 | -0.03 | 6.17E-09 | -0.03 | 1.22E-07 |
| NA* | 19 | cg19821297 | -0.02 | 1.15E-07 | -0.02 | 1.96E-08 | -0.03 | 4.16E-07 | -0.02 | 8.12E-08 | -0.02 | 1.16E-07 | -0.02 | 5.60E-08 | -0.02 | 2.87E-08 | -0.02 | 6.78E-08 |
| *SOCS3* | 17 | cg18181703 | -0.02 | 1.01E-07 | -0.03 | 3.99E-08 | -0.02 | 5.13E-07 | -0.02 | 9.70E-08 | -0.02 | 2.49E-07 | -0.02 | 1.49E-07 | -0.02 | 2.21E-08 | -0.02 | 1.66E-07 |

Significant associations between ln-transformed systemic CRP levels and ß-values of DNA methylation sites were assessed using multivariate linear mixed effects models adjusting for age, sex, BMI, fasting status, cigarette smoking, white blood cell components estimates, and the additional covariates as fixed and technical covariates as random effects. *No gene annotation for this CpG site according to the UCSC Genome Browser †For cg26470501: rs4803749,rs2965104, rs56702353, rs59927282, rs17658713, rs7257231, rs56978169, rs2927488, rs77775146, rs2965105, rs8100197, rs111252495, rs59170622, rs10422350, rs143432496, rs17728272, rs2965102, rs112524726, rs4802235, rs2965103, for cg19821297: rs76211352, rs11667764, rs56264140, rs34635891, rs3745652, rs62108388, rs6511826, rs4804729, rs896388, rs11667765, rs1205169, rs66630592. There were no SNPs with correlation coefficients ≥ 0.8 for cg02716826 and cg18181703. ‡Lipids include lipid ratio defined as total cholesterol levels divided by high-density lipoprotein cholesterol, triglycerides, and low-density lipoprotein cholesterol. §Medication includes systemic hormone therapy (yes/no/male) and other medication including, regular intake of corticoids or non- steroidal anti- inflammatory drugs, antidiabetic medication, intake of antihypertensive and lipid lowering drugs (yes/no).

## Table B. Significant associations (Benjamini Hochberg corrected) between CRP and DNA methylation sites in the KORA F4 discovery study

| **CpG** | **chr** | **gene** | **ß coef** | **se** | **p** |
| --- | --- | --- | --- | --- | --- |
| cg26470501 | 19 | *BCL3* | -0.030 | 0.005 | 6.14E-10 |
| cg02716826 | 9 | *AQP3* | -0.031 | 0.005 | 2.72E-08 |
| cg19821297 | 19 | *NA** | -0.024 | 0.004 | 5.19E-08 |
| cg18181703 | 17 | *SOCS3* | -0.023 | 0.004 | 1.01E-07 |
| cg07826859 | 7 | *MYO1G* | -0.029 | 0.006 | 1.62E-07 |
| cg10963664 | 10 | *DUSP13* | -0.033 | 0.006 | 2.30E-07 |
| cg19266329 | 1 | *NA** | -0.021 | 0.004 | 2.61E-07 |
| cg22805381 | 14 | *NA** | -0.019 | 0.004 | 2.80E-07 |
| cg19572487 | 17 | *RARA* | -0.016 | 0.003 | 3.26E-07 |
| cg12513616 | 5 | *NA** | -0.023 | 0.005 | 3.34E-07 |
| cg17501210 | 6 | *RPS6KA2* | -0.018 | 0.004 | 3.34E-07 |
| cg10919522 | 14 | *C14orf43* | -0.023 | 0.005 | 4.82E-07 |
| cg18608055 | 19 | *SBNO2* | -0.027 | 0.005 | 5.60E-07 |
| cg12170787 | 19 | *SBNO2* | -0.055 | 0.011 | 6.69E-07 |
| cg07573872 | 19 | *SBNO2* | -0.026 | 0.005 | 1.00E-06 |
| cg24890054 | 2 | *KCNS3* | 0.024 | 0.005 | 1.04E-06 |
| cg02711608 | 19 | *SLC1A5* | -0.025 | 0.005 | 1.12E-06 |
| cg21161138 | 5 | *AHRR* | -0.021 | 0.004 | 1.22E-06 |
| cg26725076 | 12 | *EP400NL* | -0.023 | 0.005 | 1.37E-06 |
| cg06762457 | 6 | *ZC3H12D* | -0.014 | 0.003 | 1.43E-06 |
| cg08548559 | 22 | *PIK3IP1* | -0.018 | 0.004 | 1.60E-06 |
| cg07094298 | 4 | *TNIP2* | -0.016 | 0.003 | 2.21E-06 |
| cg09113939 | 16 | *NA** | -0.025 | 0.005 | 2.43E-06 |
| cg22103219 | 7 | *SH2B2* | -0.019 | 0.004 | 2.57E-06 |
| cg07578976 | 6 | *PPP1R3G* | 0.029 | 0.006 | 2.82E-06 |
| cg21566642 | 2 | *NA** | -0.013 | 0.003 | 2.89E-06 |
| cg00991794 | 6 | *STL* | 0.024 | 0.005 | 2.92E-06 |
| cg05784862 | 17 | *KSR1* | -0.021 | 0.004 | 3.23E-06 |
| cg05673882 | 5 | *POLK* | -0.015 | 0.003 | 3.29E-06 |
| cg25368647 | 5 | *MXD3* | -0.017 | 0.004 | 3.61E-06 |
| cg13328614 | X | *PIM2* | -0.017 | 0.004 | 3.73E-06 |

Associations between ln-transformed systemic CRP levels and ß-values of DNA methylation sites were assessed using multivariate linear mixed effects models adjusting for age, sex, BMI, smoking, fasting status, and estimated white blood cell composition as fixed effects, and technical variables as random effects, and correcting for multiple testing according to Benjamini-Hochberg.

chr: chromosome; gene: UCSC reference gene; ß coef: β coefficient; se: standard error

*No gene annotation for this CpG site according to the UCSC Genome Browser

## Table C. Associations between CRP and DNA methylation sites in the LMU liver cohort.

| **Gene** | **Chr** | **CpG** | **ß coef** | **se** | **p** |
| --- | --- | --- | --- | --- | --- |
| *SOCS3* | 17 | CpG_2.3 | -0.04 | 0.01 | **4.36E-07** |
| *SOCS3* | 17 | CpG_8.9 | -0.03 | 0.01 | **2.82E-05** |
| *AQP3* | 9 | CpG_3 | 0.04 | 0.01 | **1.51E-03** |
| *AQP3* | 9 | CpG_2 | 0.12 | 0.04 | 5.43E-03 |
| *NA* | 19 | CpG_3 | -0.02 | 0.01 | 8.62E-03 |
| *SOCS3* | 17 | CpG_10 | -0.01 | 0.01 | 1.13E-02 |
| *SOCS3* | 17 | CpG_11 | -0.03 | 0.01 | 1.14E-02 |
| *SOCS3* | 17 | CpG_15 | -0.03 | 0.01 | 1.14E-02 |
| *NA* | 19 | CpG_5 | -0.02 | 0.01 | 1.93E-02 |
| *NA* | 19 | CpG_1 | 0.02 | 0.01 | 4.43E-02 |
| *SOCS3* | 17 | CpG_1 | -0.02 | 0.01 | 4.94E-02 |
| *SOCS3* | 17 | CpG_5 | -0.02 | 0.01 | 4.94E-02 |
| *AQP3* | 9 | CpG_5 | 0.02 | 0.01 | 5.69E-02 |
| *NA* | 19 | CpG_4 | -0.01 | 0.01 | 6.20E-02 |
| *SOCS3* | 17 | CpG_20 | -0.01 | 0.01 | 9.97E-02 |
| *AQP3* | 9 | CpG_1 | -0.06 | 0.03 | 1.09E-01 |
| *SOCS3* | 17 | CpG_4 | -0.01 | 0.01 | 1.12E-01 |
| *SOCS3* | 17 | CpG_12 | -0.01 | 0.01 | 1.93E-01 |
| *BCL3* | 19 | CpG_1 | -0.01 | 0.01 | 3.24E-01 |
| *SOCS3* | 17 | CpG_19 | 0.01 | 0.01 | 4.88E-01 |
| *SOCS3* | 17 | CpG_14 | 0.00 | 0.01 | 6.06E-01 |
| *NA* | 19 | CpG_2 | 0.00 | 0.01 | 6.53E-01 |
| *AQP3* | 9 | CpG_8.9 | 0.00 | 0.01 | 7.82E-01 |
| *AQP3* | 9 | CpG_7 | 0.00 | 0.01 | 7.94E-01 |
| *SOCS3* | 17 | CpG_16.17.18 | 0.00 | 0.01 | 8.12E-01 |
| *SOCS3* | 17 | CpG_6.7 | 0.00 | 0.01 | 9.36E-01 |

Significant associations between systemic CRP levels and ß-values of DNA methylation sites were assessed using multivariate linear models adjusting for 10 years age groups, sex, smoking, experimental plate, information on chemotherapy, and indication of operation. Significant hits after correcting for multiple testing using Bonferroni method (p < 1.92E-03) are marked in bold font.

chr: chromosome; Gene: UCSC reference gene; ß coef: β coefficient; se: standard error

*No gene annotation for this CpG site according to the UCSC Genome Browser

## Table D. Sequences of PCR tagged primers used for EpiTYPER methylation analysis, product size of each amplicon, and informative CpG sites per amplicon.

| Amplicon | Direction | Primer | Sequence | Product size (bp) | Total No. of CpG sites/ coverage in amplicon | No. of analyzed CpG sites per amplicon* | |
| --- | --- | --- | --- | --- | --- | --- | --- |
|  |  |  |  |  |  |  |  |
|  |  |  |  |  |  | Single | Composite |
| *BCL3* | forward | left | aggaagagagAGGAATTAAGATATTTGGAGATATGGG | 399 | 2 / 1 | 1 | 0 |
|  |  | right | cagtaatacgactcactatagggagaaggctAAACCCTCTATTCTTAAACTCCCAA |  |  |  |  |
| *AQP3* | reverse | left | aggaagagagAGTGATTTAGTTAATAGGTTGGGGG | 375 | 9 / 7 | 5 | 2 |
|  |  | right | cagtaatacgactcactatagggagaaggctAAACACCAAATAAACAACTTTAAACA |  |  |  |  |
| *Chr. 19* | forward | left | aggaagagagAATTTAGTAAGAGGGGTTGAAAAAT | 330 | 5 / 5 | 5 | 0 |
|  |  | right | cagtaatacgactcactatagggagaaggctCCCTAATAAAAACTAAATCCCTACCC |  |  |  |  |
| *SOCS3* | forward | left | aggaagagagGGTAGTTGGGTGATTTTTTTATAGGA | 378 | 20 / 19 | 10 | 9 |
|  |  | right | cagtaatacgactcactatagggagaaggctAACCCAATCTAAAACCAAAAACCT |  |  |  |  |

*The total number of informative CpG sites in the amplicon is divided into single CpG sites and composite sites (two or more adjacent CpG

sites fall within one fragment, or when fragment masses are overlapping).

## Table E. Primers and probes for quantitative PCRs.

| **Transcript/ NM number** | **Primer/ probe** | **Sequence** |
| --- | --- | --- |
| *AQP3* | 5’-primer | 5’-GGACCCTCATCCTGGTGAT -3’ |
| NM_004925.4 | 3’-primer | 5’-GGTTGATGGTGAGGAAACCA-3’ |
|  | probe | 5’F-TTTGGCTGTGGCTCCGTGGCC-3‘T |
| *SOCS3* | 5’-primer | 5’-AGACTTCGATTCGGGACCA-3’ |
| NM_003955.3 | 3’-primer | 5’-AACTTGCTGTGGGTGACCA-3’ |
|  | probe | 5’F-AGATCCACGCTGGCTCCGTGCG-3‘T |

Probes: 5‘F-= 5‘FAMRA-; -3‘T = -3’TAMRA


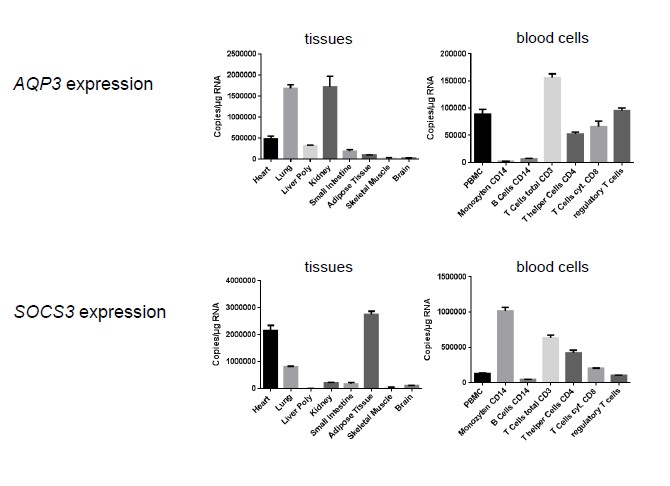


Fig A. Expression of *AQP3 and SOCS3* (normalized to μg input RNA) in different human tissues (human brain, heart, lung, kidney, small intestine, adipose tissue, skeletal muscle) and blood cell types (peripheral blood mononuclear cells (PBMC), CD14-, CD19-, CD3-, CD4-, CD8-positive cells, and regulatory T-cells).

**References**

1. Wichmann HE, Gieger C, Illig T (2005) KORA-gen--resource for population genetics, controls and a broad spectrum of disease phenotypes. Gesundheitswesen 67 Suppl 1: S26-30.

2. Bendinelli B, Masala G, Saieva C, Salvini S, Calonico C, Sacerdote C, et al. (2011) Fruit, vegetables, and olive oil and risk of coronary heart disease in Italian women: the EPICOR Study. Am J Clin Nutr 93: 275-283.

3. Fiorito G, Guarrera S, Valle C, Ricceri F, Russo A, Grioni S, et al. (2014) B-vitamins intake, DNA-methylation of One Carbon Metabolism and homocysteine pathway genes and myocardial infarction risk: the EPICOR study. Nutr Metab Cardiovasc Dis 24: 483-488.

4. Moayyeri A, Hammond CJ, Valdes AM, Spector TD (2013) Cohort Profile: TwinsUK and healthy ageing twin study. Int J Epidemiol 42: 76-85.

5. Grundberg E, Small KS, Hedman AK, Nica AC, Buil A, Keildson S, et al. (2012) Mapping cis- and trans-regulatory effects across multiple tissues in twins. Nat Genet 44: 1084-1089.

6. Thasler WE, Weiss TS, Schillhorn K, Stoll PT, Irrgang B, Jauch KW (2003) Charitable State-Controlled Foundation Human Tissue and Cell Research: Ethic and Legal Aspects in the Supply of Surgically Removed Human Tissue For Research in the Academic and Commercial Sector in Germany. Cell Tissue Bank 4: 49-56.

7. Zeilinger S, Kuhnel B, Klopp N, Baurecht H, Kleinschmidt A, Gieger C, et al. (2013) Tobacco smoking leads to extensive genome-wide changes in DNA methylation. PLoS One 8: e63812.

8. Touleimat N, Tost J (2012) Complete pipeline for Infinium((R)) Human Methylation 450K BeadChip data processing using subset quantile normalization for accurate DNA methylation estimation. Epigenomics 4: 325-341.

9. Du P, Kibbe WA, Lin SM (2008) lumi: a pipeline for processing Illumina microarray. Bioinformatics 24: 1547-1548.

10. Teschendorff AE, Marabita F, Lechner M, Bartlett T, Tegner J, Gomez-Cabrero D, et al. (2013) A beta-mixture quantile normalization method for correcting probe design bias in Illumina Infinium 450 k DNA methylation data. Bioinformatics 29: 189-196.

11. Pidsley R, CC YW, Volta M, Lunnon K, Mill J, Schalkwyk LC (2013) A data-driven approach to preprocessing Illumina 450K methylation array data. BMC Genomics 14: 293.

12. Marabita F, Almgren M, Lindholm ME, Ruhrmann S, Fagerstrom-Billai F, Jagodic M, et al. (2013) An evaluation of analysis pipelines for DNA methylation profiling using the Illumina HumanMethylation450 BeadChip platform. Epigenetics 8: 333-346.

13. Grundberg E, Meduri E, Sandling JK, Hedman AK, Keildson S, Buil A, et al. (2013) Global analysis of DNA methylation variation in adipose tissue from twins reveals links to disease-associated variants in distal regulatory elements. Am J Hum Genet 93: 876-890.
